# Effect of Walnut-Shell Additive on the Structure and Characteristics of Concrete

**DOI:** 10.3390/ma16041752

**Published:** 2023-02-20

**Authors:** Alexey N. Beskopylny, Sergey A. Stel’makh, Evgenii M. Shcherban’, Levon R. Mailyan, Besarion Meskhi, Alexandr A. Shilov, Andrei Chernil’nik, Diana El’shaeva

**Affiliations:** 1 Department of Transport Systems, Faculty of Roads and Transport Systems, Don State Technical University, Rostov-on-Don 344003, Russia; 2 Department of Unique Buildings and Constructions Engineering, Don State Technical University, Rostov-on-Don 344003, Russia; 3 Department of Engineering Geology, Bases, and Foundations, Don State Technical University, Rostov-on-Don 344003, Russia; 4 Department of Life Safety and Environmental Protection, Faculty of Life Safety and Environmental Engineering, Don State Technical University, Rostov-on-Don 344003, Russia

**Keywords:** concrete, sustainable concrete, walnut shell

## Abstract

The partial replacement of the mineral components of concrete with natural renewable analogues in full possession of the performance characteristics of the final material, allows not only the concrete-production process to be made more environmentally friendly and inexpensive, but also to solve an important task for the agricultural industry, which is that associated with waste disposal. The scientific novelty of the work is in the obtaining of new concrete compositions by the partial replacement of coarse aggregate with a natural analogue in the form of a walnut shell, which has the maximum ratio of the strength of the composite to its density, as well as in identifying new dependencies of strength and density and their ratio on the amount of replacement of mineral coarse-aggregate walnut shell. The main goal of this article was to analyze the effect of composition factors on characteristics of concrete with partial replacement of large aggregates with walnut shells and to search for the optimal compound that would make it possible to obtain concrete with a minimum decrease in strength characteristics with a maximum decrease in concrete density. Cubes and prism laboratory samples were made from concrete of normal density with the replacement of coarse aggregate by 5, 10, 15, 20, 25 and 30%, by volume. The main mechanical properties, such as density, strength (compressive, tensile, tensile strength in bending) of the concrete samples were studied. The investigation used standard methods and scanning electron microscopy. An increase into strength characteristics up to 3.5%, as well as the maximum ratio of strength to density of concrete, was observed at a walnut-shell dosage of 5%. Effective partial replacement of coarse aggregate with walnut shells leads to a reduction in the consumption of crushed stone by up to 10% and a decrease in the mass of concrete by up to 6%.

## 1. Introduction

The growth in global-construction volume is directly related to the growth in the production of building structures and, as a result, to the growing demand for building materials [1]. Concrete and reinforced-concrete structures are among the most resource-intensive, but at the same time one of the foundations of modern construction. Ordinary concrete, which is widely used as a material for building structures around the world, is a material that has been proven by many years of application practice. Despite their large reserves in nature, the growth in the production of concrete and reinforced-concrete structures can create a significant risk of their depletion. It is also important to note that the processes of extraction and production of concrete-mix components are associated with rather high energy consumption and significant greenhouse-gas emissions [2]. Given the above, the construction industry is faced with the question of finding technologies that would reduce the cost of concrete, make its production processes more environmentally friendly and reduce the need for non-renewable components of the concrete mixture [3].

In addition, in the agricultural industry there are a number of problems associated with the disposal of waste from production processes, the volume of which is growing every day [4,5]. The processes of disposal of these wastes associated with their secondary processing do not require the volume of raw materials that are generated in agricultural processes, as a result of which plant wastes are either buried, which leads to leaching of toxic chemical compounds due to decomposition processes, or are burned, which leads to large volumes of greenhouse-gas emissions and dispersion in the atmosphere of microscopic particles of combustion products [6,7]. At the same time, many such wastes from agricultural processes have strength characteristics (SC) comparable to the characteristics of the mineral components of concrete, which makes them potentially one of the main candidates for partial replacement of aggregates traditionally used for concrete [8]. Moreover, the structural features of organic materials could make it possible to obtain lighter concrete with improved sound- and heat-protection characteristics [9]. Natural coarse aggregates were replaced with recycled concrete aggregates as one solution for reducing the depletion of natural resources in various concrete applications [10,11]. Thus, the use of such agricultural by-products as a partial replacement for the components of the concrete mixture will allow not only the establishment of a large-scale, full-fledged and environmentally friendly process of recycling agricultural-production waste, but also a significant reduction in the cost of concrete, as well as a reduction in the use of non-renewable concrete components, due to their replacement with rapidly renewable natural analogues, which corresponds to the concept of sustainable development [12,13,14,15].

**Figure 1:**
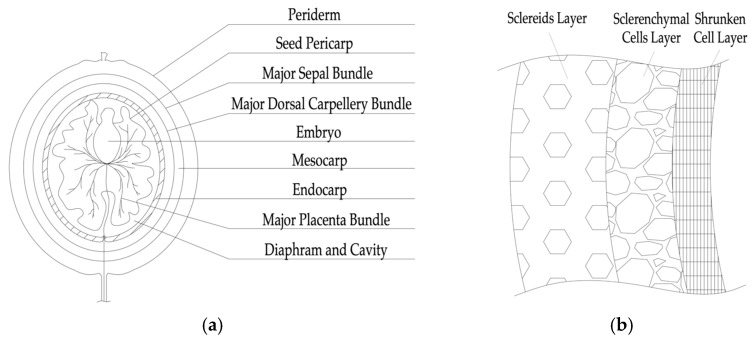
Walnut: (**a**) fruit structure; (**b**) schematic diagram of the microstructure of a hard shell.

One of the main candidates for replacing coarse aggregate in concrete is machined walnut shells. The walnut shell is a byproduct of the production of shelled walnuts, produced on a huge scale and sold all over the world. The fruit of the walnut is a pseudo-monomeric drupe-type nut. The lignified endocarp is a strong spherical or ovoid bone with several (from two to five) incomplete partitions, inside of which there is an edible seed. It is enclosed in a fibrous mesocarp, topped with a leathery exocarp. When ripe, the peel of the fruit dries up and bursts into two parts, separating from the stone, which does not open by itself [16,17,18]. The structure of the walnut fruit is shown in Figure 1a. The shell microstructure consists of three layers of different levels of porosity, from the most porous on the inside to the densest on the outside—the scleroid layer, the layer of sclerenchymatous cells, and the wrinkled-cell layer [17]. A diagram of the microstructure of a walnut shell is shown in Figure 1b.

Almost all walnut-processing enterprises experience difficulties with the sale and disposal of the shell. Along with this, the walnut shell has a sufficiently high strength, does not rot, practically does not lose its properties over time, quickly renews in nature and is an environmentally friendly and safe product for humans and animals [16]. The hard shell of a walnut consists of ash (3.4%), lignin (50.3%), hemicellulose (22.4%), and cellulose (23.9%) [16].

To create the most massive need for walnut shells, which could allow large-scale and environmentally safe disposal of its entire volume, is possible only by integrating the recycling process into large and fundamental industrial sectors. The most important candidate for this is the construction industry and the production of building structures [19]. The walnut shell, in comparison with other agricultural waste, has more valuable technical indicators. Due to the high content of lignin, its strength and resistance to decay are much better than that of straw, husks, sawdust and others. In addition, the walnut shell has the lowest water absorption among the listed materials [20]. Analyzing the above, we can confidently say that the walnut shell is a potentially interesting material for use as a partial replacement for coarse mineral aggregate in concrete.

**Figure 2:**
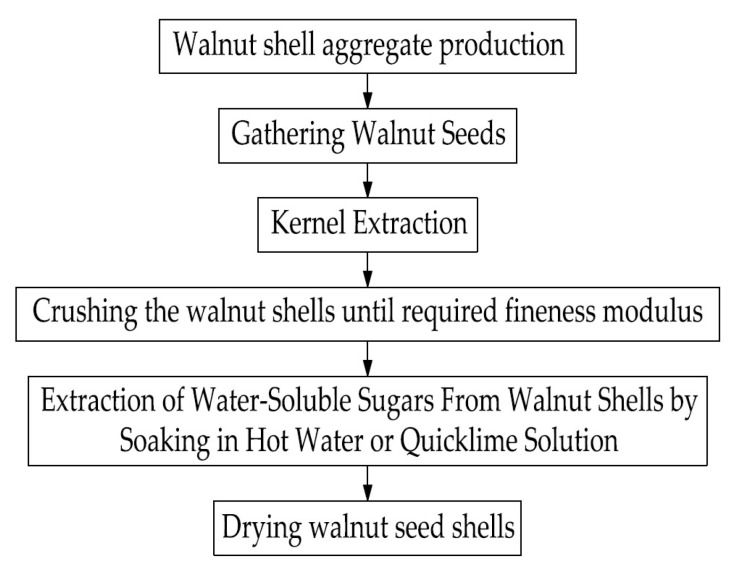
Walnut-shell-processing process.

In order to use the walnut shell as a coarse aggregate in concrete, it is first necessary to subject it to mechanical and chemical treatment. It is necessary not only to crush it to a fraction of the required size modulus, but also to keep the resulting material in hot water to extract water-soluble sugars, which can adversely affect the cement-hydration process. For these purposes, soaking quicklime in solution is also used, but it requires more time and additional costs for quicklime, which makes this method less effective [17,18,21,22]. The process of processing the walnut shell is shown in Figure 2.

Analyzing the process of processing walnut shells to obtain organic coarse aggregate for concrete, we can conclude that, despite the use of water in it and taking into account the potential energy costs for its heating, it is much more environmentally friendly and less energy-intensive than the production of a mineral coarse-aggregate placeholder. It is also important to note that, as mentioned above, the walnut shell is a rapidly renewable natural raw material for which the issue of mass disposal has not been resolved, while mineral aggregates are exhaustible and non-renewable resources that require energy-intensive and environmentally unsafe technological processes for production [23,24,25,26,27,28]. The work of the authors [21] is devoted to the study of the mechanical properties of concrete with the partial replacement of coarse aggregate with walnut shells and partial replacement of fine aggregate with metallurgical slag. The degree of replacement of fine aggregate varied (from 0% to 50%), and the degree of replacement of coarse aggregate with walnut shell remained fixed (20%). It was concluded that, despite a slight decrease in strength when walnut shells are added, this concrete is suitable for use in modern construction, and meets all the requirements. The authors also noted that such concrete has a lower weight compared to conventional concrete and exhibits higher resistance to acids and sulfates.

The study [22] was devoted to the properties of lightweight shotcrete with the addition of polyethylene terephthalate fibers and the replacement of coarse aggregate with walnut shells. The authors concluded that the complete replacement of coarse aggregate with walnut shells reduces the compressive strength (CS), flexural tensile strength (TS), and axial tension, as well as reducing settlement and pressure losses during mixture pumping. The addition of fibers somewhat leveled the loss in strength. It was concluded that the mixture with the addition of polyethylene terephthalate fibers has optimal performance at a content of coarse aggregate in the form of walnut shells in the amount of 35% by weight, and such a mixture is suitable for use as lightweight shotcrete for cast-in-place road-tunnel structures.

In [29], the authors studied the properties of self-compacting concrete with varying degrees of partial replacement of coarse aggregate with walnut shells. The SC of self-compacting concrete decreased in proportion to the increase in the amount of organic coarse aggregate. However, lightweight self-compacting concrete with characteristics that meet the requirements of modern construction can be obtained with a walnut-shell content of 35% or more. Comparing the mechanical properties of the composition with organic coarse aggregate, a decrease in strength indicators will inevitably be observed compared to concretes of normal density; however, such a composition acquires properties characteristic of lightweight concretes—lighter weight, and improved soundproof and heat-shielding properties [19,30]. The SC of the walnut shell are much higher than those of the aggregates of light concretes, which makes it possible to use it as a partial replacement for coarse aggregates in concretes of normal density, giving them properties uncharacteristic of such concretes, with minimal losses in strength indicators [31,32,33,34,35].

The study presented in this article develops the ideas of the concept of sustainable environmentally friendly concrete, which implies the partial and complete replacement of the mineral components of concrete with natural and rapidly renewable analogues. Replacing large-mineral aggregate with walnut shells will partially eliminate complex mechanized and energy-intensive production processes associated with high greenhouse-gas emissions [36,37,38,39,40,41,42]. At the same time, it is important to find a balance between the strength and density of the resulting composite, so that a decrease in the SC of the material is accompanied by a commensurate decrease in the density of the material. Thus, the scientific novelty of the work is the obtaining of new concrete compositions with partial replacement of coarse aggregate with a natural analogue in the form of a walnut shell which has the maximum ratio of the strength of the composite to its density, as well as in identifying new dependencies of strength, density and their ratio on the amount of replacement of mineral coarse-aggregate walnut shell.

Therefore, the main goal of the work was to study the effect of partial replacement of coarse mineral aggregate with mechanically processed and prepared-for-use walnut shells on the structure and characteristics of concrete and the selection of the optimal amount of walnut shells, which would make it possible to obtain an environmentally friendly building material with the highest ratio of strength to density.

## 2. Materials and Methods

### 2.1. Materials

Raw materials such as Portland cement, crushed stone, and sand and walnut shells were used to make concrete samples. The main physical and mechanical characteristics of raw materials are presented in Table 1 and Table 2.

The mineral content of Portland cement is as follows: 67% of C_3_S, 15% of C_2_S, 7% of C_3_A and 11% of C_4_AF.

**Figure 3:**
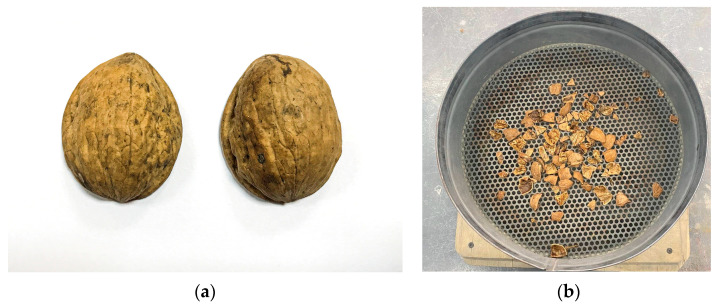
Appearance of the walnut shell: (**a**) whole; (**b**) crushed to sizes from 5 to 20 mm.

The walnut shell underwent the necessary mechanical processing to achieve a grain size of 5 to 20 mm, and was purified from water-soluble sugars. Shell cleaning was carried out in water at a temperature of 60 °C, in which it was kept for 24 h for better extraction of water-soluble sugars. Then, the water with dissolved sugars was drained and the walnut shell was dried in an oven at a temperature of 50–55 °C. The content of the fraction from 5 mm to 10 mm was 70.4%. A photograph of a walnut shell is shown in Figure 3.

### 2.2. Methods

The production of concrete samples was carried out in laboratory conditions using concrete of normal density class B30 (CS ranging from 37.5 MPa to 50 MPa), according to GOST 26633-2015 [43]. The concrete-mix formulation is shown in Table 3.

Mixing of concrete components was carried out using a BL-10 laboratory concrete mixer (ZZBO, Zlatoust, Russia). Vibration of the concrete of the samples during their manufacture was carried out on the vibrating platform SMZh-539-220A (IMash, Armavir, Russia), and the vibration time was 60 s. Testing of prototypes was carried out using the following equipment: testing machine P-50 (PKC ZIM, Armavir, Russia), tensile testing machine R-50 (IMash, Armavir, Russia).

The walnut shell was crushed on the ShchD-6 jaw crusher manufactured by Vibrotechnik (St. Petersburg, Russia). Grains less than 5 mm and more than 20 mm were screened out using a set of sieves for coarse aggregate with mesh sizes of 5, 10 and 20 mm, manufactured by Vibrotekhnik (St. Petersburg, Russia). The walnut shell was dried in an oven SNOL 67/350 (Umega, Ukmerge, Lithuania).

The production of the concrete mixture, molding, demolding and hardening of the samples were carried out according to the method already described by the authors earlier in the work [45]. The walnut shell was added to the concrete mixture after water, cement, sand and crushed stone at the end. The mixing of the components in the concrete mixer was carried out until a mixture of a homogeneous consistency was obtained. Then, the mixture was poured into metal molds for samples, which were installed on a laboratory vibration platform and compacted by vibration to the required state. The samples hardened for 28 days in a normal hardening chamber. After 24 h of curing, the samples were removed from the molds and placed back into the chamber. The samples were subjected to a compression test, bending-tensile and axial-tensile tests, in accordance with the requirements of GOST 10180 [46] and axial-compression GOST 24452 [47]. Statistical processing of the results of calculating the strength was carried out according to GOST 18105-2018 [48].

**Figure 4:**
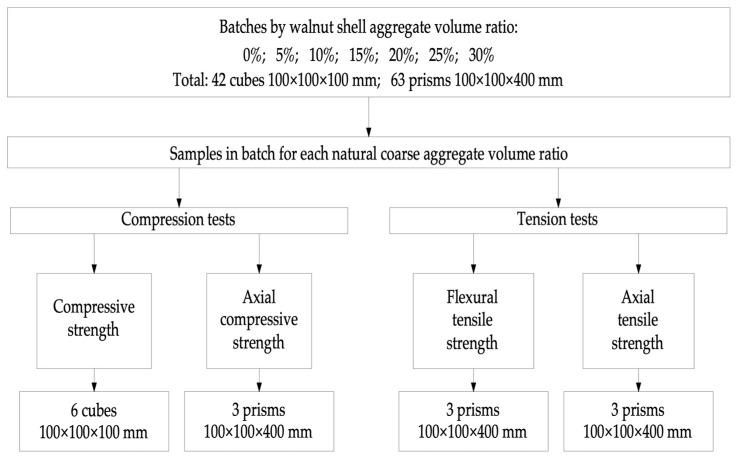
Program for testing concrete samples.

The microstructure of the samples was analyzed using a ZEISS CrossBeam 340 electron microscope (Carl Zeiss Microscopy GmbH (Factory), Jena, Germany), as in [45]. Seven series of samples were prepared with different values of substitution of coarse mineral aggregate, with a vegetable analogue from walnut shell. By the term “vegetable analogue”, the authors mean a promising and effective component for the concrete mixture, which is of plant origin. In the case of the use of walnut shells, such a vegetable analogue makes it possible to partially replace such a concrete component as an aggregate. The program for testing the concrete samples is shown in Figure 4.

Thus, for the compression test, 42 cubes were made (6 cubes for each mixture), for axial compression—21 prisms (3 prisms each), for bending—21 prisms (3 prisms each), and for axial tension—21 prisms (3 prisms each).

The purpose of axial CS test is to correct and verify the CS. As a rule, cubic strength is a standardized normative concept that characterizes the class of concrete as a building material. However, a number of normative and technical sources in the territory of the CIS countries and some countries of Eurasia also recommend the use of an additional indicator, “axial compressive strength”, if it is necessary, to calculate reinforced-concrete structures and determine precisely the structural characteristics of concrete.

The indicator characterizing the ratio of the CS of concrete to its density, called the coefficient of constructive quality (CCQ), was calculated by the formula:

(1) $$\mathrm{CCQ}=\frac{R_{b.\mathrm{cub}}}{\rho }$$

where *R*_b.cub_—compressive strength determined on concrete cube samples; *ρ*—density of concrete samples.

## 3. Results and Discussion

### 3.1. Study of the Physical and Mechanical Characteristics of Concrete

**Figure 5:**
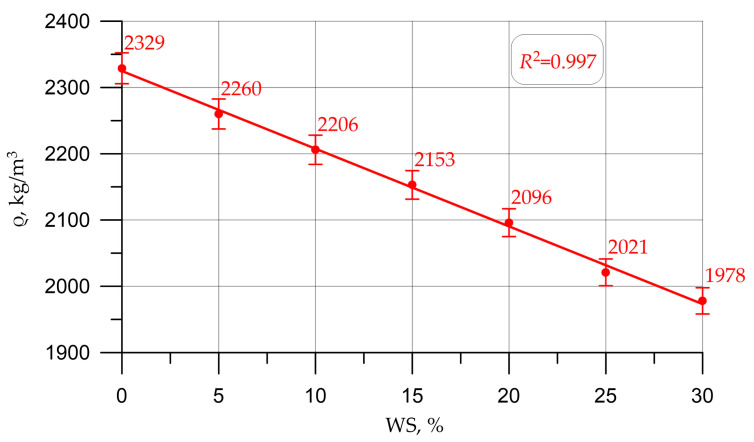
Dependence of concrete density on the amount of walnut shell.

**Figure 6:**
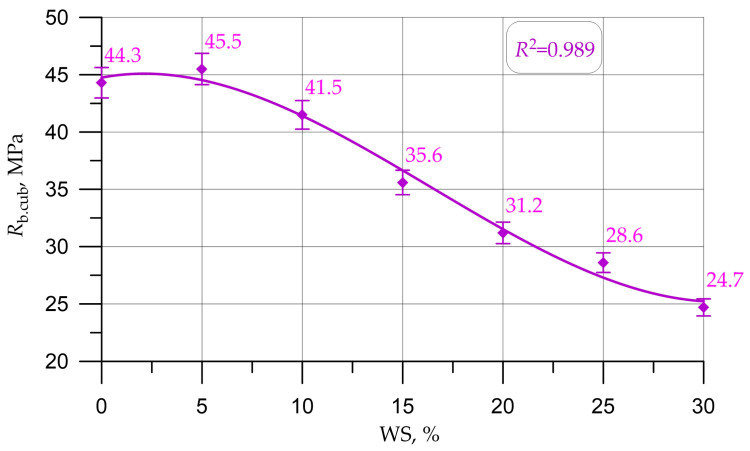
Dependence of the cubic strength of concrete on the amount of walnut shell.

**Figure 7:**
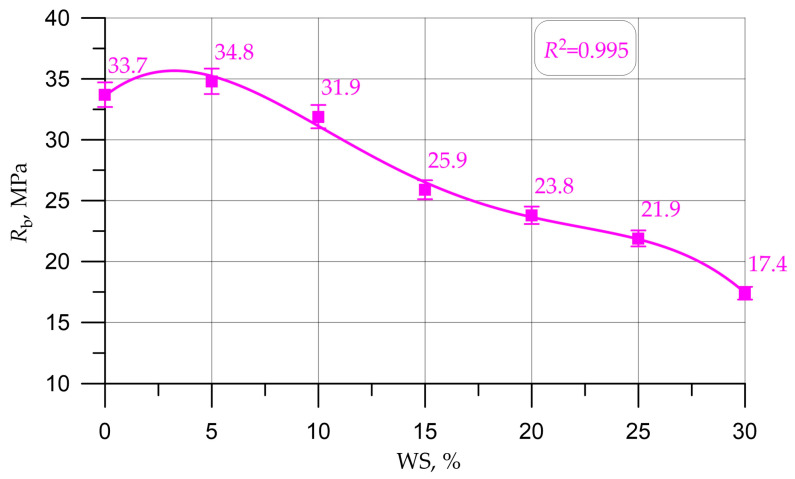
Dependence of the prism strength of concrete on the amount of walnut shell.

**Figure 8:**
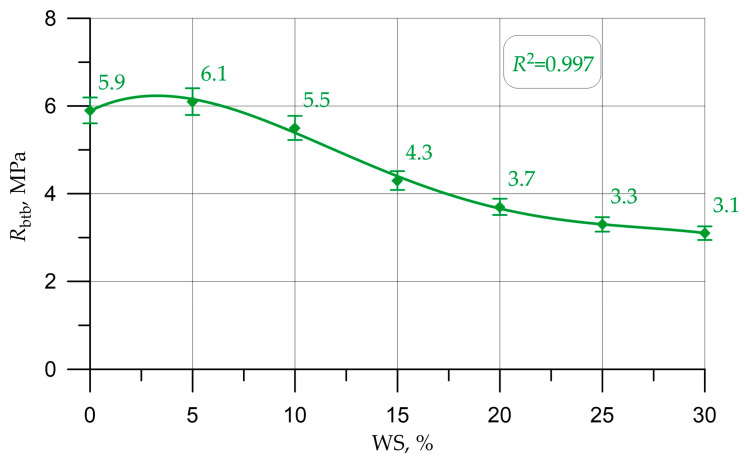
Dependence of the TS in bending of concrete on the amount of walnut shell.

**Figure 9:**
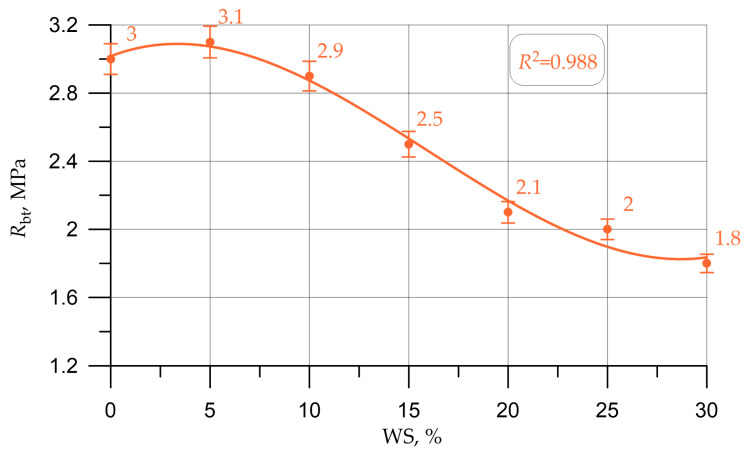
Dependence of the axial TS of concrete on the amount of walnut shell.

Graphs in Figure 5, Figure 6, Figure 7, Figure 8 and Figure 9 illustrate dependencies characterizing the influence of the percentage of replacement of large mineral aggregate with walnut shells on the physical and SC of concrete: density (Figure 5), cubic strength (Figure 6), prism strength (Figure 7), TS in bending (Figure 8), and axial TS (Figure 9).

The dependencies presented in Figure 5, Figure 6, Figure 7, Figure 8 and Figure 9 are well approximated by polynomial dependencies of various degrees, and are shown in Equations (2)–(6), with a determination coefficient *R*^2^

(2) $$\rho =2324.8\;-\;11.72\;x,\;R^{2}=0.997$$

(3) $$R_{b.\mathrm{cub}}=44.75+0.33\;x\;-\;0.0834\;x^{2}+0.001689\;x^{3},\;R^{2}=0.989$$

(4) $$R_{b}=33.60+1.393\;x\;-\;0.273\;x^{2}+0.01286\;x^{3}\;-\;0.000197\;x^{4},\;R^{2}=0.995$$

(5) $$R_{\mathrm{btb}}=5.88+0.23\;x\;-\;0.0435\;x^{2}+0.00178\;x^{3}\;-\;2.303\times 10^{-5}\;x^{4},\;R^{2}=0.997$$

(6) $$R_{\mathrm{bt}}=3.016+0.045\;x\;-\;0.00747\;x^{2}+0.155\times 10^{-3}\;x^{3},\;R^{2}=0.988$$

Discussing the resulting graphs in Figure 5, Figure 6, Figure 7, Figure 8 and Figure 9, and considering them individually, one should note their common features, while at the same time some differences. Therefore, from the resulting concrete-density graph, a tendency to a gradual decrease in density, and, consequently, the weight of concrete and structures made from it, is clearly seen. Furthermore, analyzing the strength graphs, it can be seen that, to one degree or another, the increase in concrete strength was maximum for all types of strengths at a dosage of walnut shells in the amount of 5%. Comparing the density graph and the strength graphs with each other, having additionally built and calculated graphically and mathematically the CCQ, the thesis regarding the rationality and effectiveness of the walnut-shell dosage in the amount of 5% is confirmed.

Changes in the characteristics of concrete depending on the amount of mineral coarse aggregate replaced by walnut shell are shown in Table 4 and are presented as a percentage compared to the control composition, that is, conventional concrete without walnut shell.

According to the test results presented in Figure 5, Figure 6, Figure 7, Figure 8 and Figure 9 and Table 4, it was found that the best values for mechanical characteristics are observed in concrete with a walnut-shell content of 5%, introduced instead of a part of the mineral coarse aggregate. Compared to the control formulation, CS increased by 2.7%, axial CS increased by 3.3%, flexural TS increased by 3.4%, axial TS increased by 6.7%, and density decreased by 3.0%. Such increases in characteristics allow us to state that, with a carefully selected composition of concrete with the addition of a walnut shell in a certain amount, it is possible to even slightly improve the mechanical properties of concrete, and not only maintain them at the same level, as in [26,27,28,29]. The increase in SC can be explained by the fact that the crushed walnut shell, introduced instead of a part of the mineral aggregate in an amount of 0 to 5%, acts as an additional sealing element. Crushed nutshell has grains ranging in size from 5 to 20 mm. At the same time, most of these grains (more than 70%), having sizes from 5 to 10 mm, are characterized by an angular shape and a rough surface. The angular shape and rough surface are structural and technological advantages, due to the good adhesion of such components to the cement stone, which leads to an improved structure and high technological properties of the created concrete composite. Thus, in the process of manufacturing a concrete mixture and its further compaction, walnut-shell grains are located between larger grains of the mineral composite and additionally compact the composite structure at the cement-sand-mortar–coarse-aggregate phase boundary. First of all, this compaction effect is due to the fact that, due to the more angular, rough structure, the aggregate grains from the walnut shell attract a larger amount of the mortar-binder part, which also follows from the study [17].The technological feature of introducing the walnut shell into the formed concrete mix is the following: First, crushed stone is directly introduced into the mixture of water, binder and fine aggregate. Then, walnut shells are introduced into the continuously stirred said mixture, and the said mixture is mixed until it reaches the most homogeneous state.

**Figure 10:**
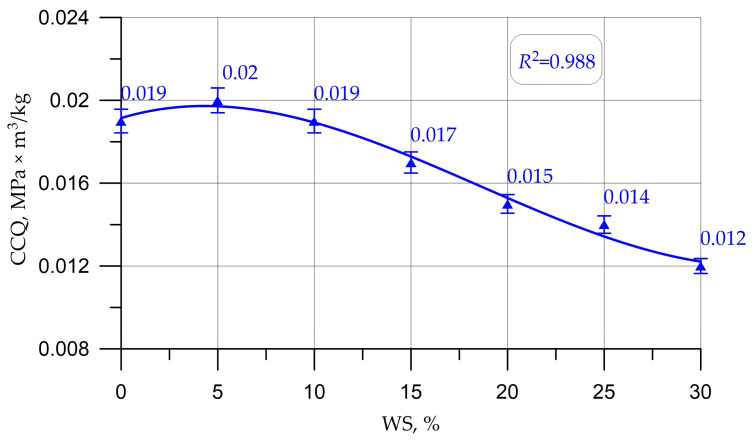
Change in the CCQ of concrete depending on the walnut-shell content.

However, a further increase in the percentage of replacement of coarse aggregate with a vegetable analogue led to a decrease in SC. This trend is due to the fact that the properties of the walnut shell are closer to coarse aggregates for lightweight concrete and the partial replacement of the mineral aggregate in heavy concrete with walnut shell will inevitably lead to a certain degree of reduction in SC, which is in good agreement with the works [21,22,25,29], which show a similar trend. However, the weight of concrete also decreases and, accordingly, the CCQ of concrete increases. Therefore, based on the results of determining the density and CS of concretes with different percentages of replacement of large mineral aggregate with walnut shells, the CCQ of concrete were calculated (Figure 10).

The best value for the CCQ is for concrete with a walnut-shell content of 5%, and the CCQ values for concrete with the control composition and with 10% walnut-shell content are approximately equivalent. The dependence of CCQ on the WS content is well approximated by a polynomial of the third degree, and is shown in Equation (7) with a determination coefficient

(7) $$\mathrm{CCQ}=0.0191+0.283\times 10^{-3}x-\;3.714\times 10^{-5}x^{2}+6.67\times 10^{-7}x^{3},\;R^{2}=0.988$$

The analysis of the results of experimental studies of the characteristics of concrete samples with the partial replacement of crushed stone with walnut shells, including plotted-graphical dependencies and data on the effect of the amount of coarse aggregate from walnut shells in concrete on its physical and mechanical characteristics, as well a comparison of the results obtained with the results obtained on the control composition, found that not only the presence of organic coarse aggregate, but also its quantity in relation to the volume of coarse mineral aggregate, significantly affected the characteristics of concrete. It was found that the optimal amount for replacing dense aggregate with walnut shells is 5%, which roughly coincides with [25,28], where the optimal dosage was up to 5%. With this percentage of replacement, concrete has the best SC and strength-to-density ratio. It was also found that a slight decrease in the SC of concrete was observed before increasing the dosage of crushed walnut shells from 5% to 10%. With an increase in the amount of WS from 10% to 30%, a more rapid drop in strength was observed. Thus, it has been determined that partial replacement of coarse mineral aggregate with walnut shells in an amount of 5% to 10% of the volume of coarse mineral aggregate makes it possible to obtain a concrete SC approximately equivalent to the SC of concrete of the control composition and within the same CS class B30, and is optimal. The value of the cubic strength in this case was 45.5 MPa for 5% replacement and 41.5 MPa for 10%, the axial CS was 34.8 MPa for 5% and 31.9 for 10%, the TS in bending was 6.1 MPa for 5% and 5.5 MPa for 10%, and the axial TS was 3.1 MPa for 5% and 2.9 MPa for 10%.

### 3.2. Analysis of the Microstructure of Samples of Hardened Cement Paste with Partial Replacement of Crushed Stone with Walnut Shells

**Figure 11:**
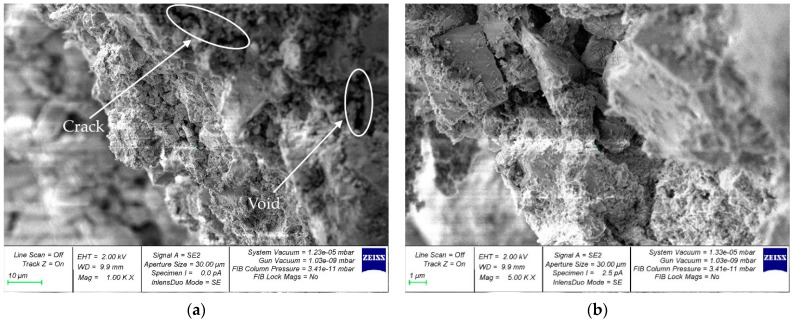
Micrographs of the structure of samples of the hardened cement paste of the control composition (containing no walnut shell): (**a**) 1000× (**b**) 5000×.

**Figure 12:**
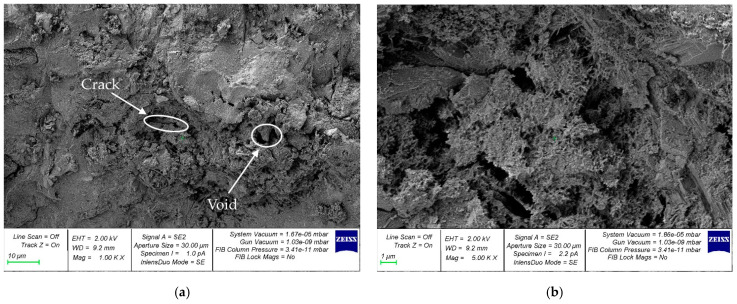
Micrographs of the structure of samples of hardened cement paste with partial replacement of mineral coarse aggregate with walnut shell, in the amount of 5%: (**a**) 1000× (**b**) 5000×.

**Figure 13:**
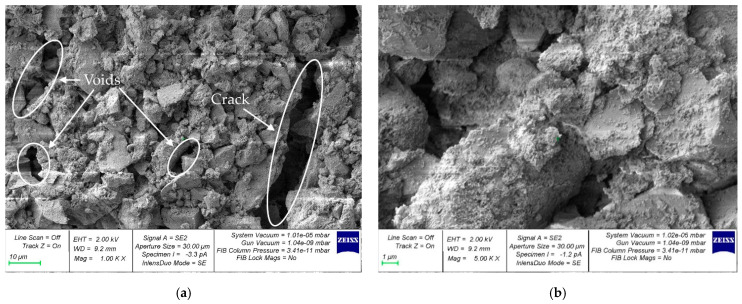
Micrographs of the structure of the hardened cement paste with partial replacement of mineral coarse aggregate with walnut shell, in the amount of 30%: (**a**) 1000×; (**b**) 5000×.

For the study of the microstructure, samples of hardened cement paste were selected at the boundaries of the contact zones with coarse aggregate. Figure 11, Figure 12 and Figure 13 show micrographs of samples of the hardened cement paste of the control composition (Figure 11) and with a walnut-shell content of 5% (Figure 12) and 30% (Figure 13), which showed the best and worst values of SC, respectively.

Figure 12 shows that the microstructure of hardened-cement-paste samples with partial replacement of coarse mineral aggregate with walnut shell in an amount of 5% has fewer voids and microcracks compared to the control composition (Figure 11), which in turn is confirmed by its better SC. Figure 13 shows that the microstructure of hardened-cement-paste samples taken from concrete with a walnut-shell content of 30% has a large number of voids and microcracks. The formation of microcracks along the area of contact between the cement gel and the organic aggregate is primarily due to the higher water absorption of the walnut shell, compared to the mineral aggregate. As a result, a greater number of microcracks at the boundaries of the contact zone, the hardened-cement-paste—coarse-aggregate, reduces the anchoring of the walnut shell in concrete and its SC.

However, with a rational dosage, the replacement of a part of the mineral coarse aggregate with an organic one positively affects the SC of the composite (5%) or leads to a slight decrease (10%), while reducing the weight of the final composite material. Thus, the addition of walnut shells to concrete as an alternative to coarse mineral aggregate is acceptable to lighten the weight of concrete and reinforced concrete structures and to dispose of waste in the form of walnut shells.

### 3.3. Discussion

The results obtained during the study are important for construction practice, but they need to be explained and discussed according to the scientific fundamental principle of a composition-structure characteristics. Thus, in the course of the experiment, increments, or the preservation of the values of the CCQ at the proper level, were established, due to the fact that the study analyzed lightweight concretes, that is, those having a reduced mass, and thereby expanded their universal operational ability.

Let us analyze the obtained results in the context of scientific justification. Due to the fact that the walnut shell has an advantageous rough surface which is similar to that of low-density crushed stone, and, in principle, by its very nature is similar to the interaction inside concrete of those concrete components with lightweight aggregates, such a walnut and its shell can be compared to these aggregates. The increased roughness and angularity of some forms allow them to achieve better adhesion of such an aggregate with hardened cement paste. This provides better structure formation and improves the formation of the composite at the stage of hardening, which ultimately leads to a more perfect structure and a sufficiently high mechanical strength inherent in such lightweight concretes. It is the rational ratio of strength and density properties in such concretes that ensures the creation of a high CCQ. This is confirmed not only by experimental tests, but also by microscopic studies using the method of scanning electron microscopy. Thus, the applied CCQ is confirmed by the fundamental principle of a better structure, from which high-performance properties follow, and all this is initially justified by a rational choice of composition. Of course, an inefficient dosage of walnut shells can lead to a deterioration in mechanical properties and structure in the cases below. With an excessive dosage of walnut shells, due to its roughness, the need for additional wetting of the surface of such a filler will occur, which will lead to excessive water consumption. Such a factor will lead to a deterioration in the structure and, accordingly, the properties of concrete. If we assume the insufficient introduction of walnut shell in an amount less than rational, then this may lead to the under-formation of the proper structure in terms of a deterioration in operational properties, that is, an unreasonable increase in the weight of the structure and a discrepancy between its operational density in relation to its strength. Thus, in order to achieve an effective balance between structural perfection and operational properties, one should strictly adhere to the experimentally established rational dosage of walnut shells used as aggregate for lightweight concrete, with an increased CCQ. This is in good agreement with the works [26,27,28].

In [22], various types of waste were used in shotcrete technology, including walnut shells, and an increase in the dosage of walnut shells also negatively affected the SC of concrete, as in this study. However, with a suitable dosage of 35% walnut shells, segregation was reduced and the flow of fresh concrete was improved. In [29], in general, the nature of the change in SC depending on the percentage of replacement of mineral aggregate with organic aggregate is the same as in this study: the greater the percentage of replacement, the greater the drop in SC. In general, the optimal dosage of the walnut- shell content in lightweight structural concrete is 35%, and the SC are within acceptable limits. So, for example, in [25], when replacing coarse mineral aggregate with walnut shell, starting from 5% and up to 25% of the replacement, a drop in SC and a decrease in density were observed. In this study, on the contrary, at 5% replacement a slight increase in SC is observed; however, a further decrease in SC shows a similar trend to that in the study [25], although the intensity of the decrease in CS in this study is less.

Analyzing the above, we can conclude that the characteristics of the walnut shell make it possible to use it as a partial replacement for the mineral coarse aggregate of light and heavy concrete for the production of concrete and reinforced-concrete structures, small architectural forms, and elements of decoration, decor and landscaping. For the construction industry, this would significantly reduce the cost of concrete, as well as from avoid a number of processes that pollute the environment, while almost completely preserving the strength and physical and mechanical characteristics of concrete. From the point of view of the agricultural industry, this would allow for the mass environmentally friendly, efficient and sustainable disposal of large volumes of waste. This also corresponds to the idea of introducing environmentally friendly “green” technologies into production processes, which provide for waste-free production and the use of natural, environmentally friendly, completely renewable materials that are safe for humans and animals. Thus, the presented work is both of an applied technological nature and opens up prospects for further research in the field of economic efficiency and ecology.

## 4. Conclusions

Based on the study, the following conclusions were made.

(1) In the course of the study, the main parameters and characteristics of the organic large-walnut-shell aggregate used to partially replace the mineral components of the concrete mixture, were determined.
(2) According to the results of the experiments, the dependences of the physical and mechanical characteristics of concrete on the amount of added walnut shell and the microstructure of the composite, were established. Improving the properties of concrete, due to the partial replacement of coarse mineral aggregate with a vegetable analogue from walnut shells, is expressed in a decrease in its weight and an increase in the CCQ. The maximum increase in strength parameters for concrete with 5% replacement of a part of the mineral coarse aggregate was: for cubic CS—2.7%, for axial CS—3.3%, for TS in bending—3.4%, and for axial TS—3.3%. The maximum decrease in the weight of concrete, at which a decrease in SC of no more than 10% was observed, reached 6%.
(3) The optimal percentage for the replacement of mineral coarse aggregate with walnut shell was in the range of 5% to 10%. Increasing the CCQ in this case amounted to 6%.
(4) An increase in the dosage of walnut shells from more than 10% to 30% leads to a decrease in SC of up to 45%, in weight of up to 17% and in CCQ of up to 37%.
(5) The study of the resulting composition using electron-microscopy methods showed that the resulting concrete composition is effective both in terms of the characteristics of the hardened composite and in terms of its economic and environmental efficiency, compared to the control composition of concrete without the introduction of walnut shells.

The significant advantage of the proposed composition of concrete for economics and ecology, while maintaining its overall strength compared to conventional concrete, will open up broad prospects for further research and practical application. The presented work has an applied technological character and also opens up prospects for further research in the fields of economic efficiency and ecology.

## Figures and Tables

**Table 1:** Characteristics of the binder.

Material Name	Specific Surface (m^2^/kg)	The Normal Density of Cement Paste (%)	Fineness Grinding, Passage through a Sieve No. 008 (%)	Setting Time (min)	Tensile Strength in Bending (MPa)	Compressive Strength (MPa)
CEM I 52.5 N produced by JSC MORDOVCEMENT (p. Komsomolsky, Russia)	330	25	4.3	start 165 end 230	4.5 (2 days) 7.8 (28 days)	24.3 (2 days) 55.7 (28 days)

**Table 2:** Characteristics of aggregates.

Aggregate’s Title	Actual Value
Quartz sand produced by Nedra (Samarskoye, Russia)	
Size modulus	1.73
Bulk density (BD) (kg/m^3^)	1578
True density (TD) (kg/m^3^)	2650
The content of dust and clay particles (%)	1.1
Clay content in lumps (%)	0.15
Crushed sandstone produced by Aninsky GOK (Anikin farm, Russia)	
Fraction size (mm)	from 5 to 20
BD (kg/m^3^)	1487
TD (kg/m^3^)	2655
Crushability (% by mass)	10.6
The content of lamellar (flaky) and needle-shaped grains (% by weight)	9.8
Walnut shell (collected in Krasnodar region, Russia)	
Flakiness index, FI (wt. %)	91.15
Relative density (SSD)	1.45
Water absorption (wt, %)	18.11
BD (kg/m^3^)	545
Dry-air humidity (wt.%)	2.01
Crushability (% by mass)	0.98
The content of grains of lamellar (flaky) and acicular form (% by weight)	48.23

**Table 3:** The concret-mix formulation.

Component Name	Portland Cement (kg/m^3^)	Water (L/m^3^)	Crushed Stone (kg/m^3^)	Sand (kg/m^3^)	Walnut Shell, (kg/m^3^)	ρ_cm_ (kg/m^3^)	Cone Draft According to GOST 10181 [44]
Amount per 1 m^3^ of concrete mix	375	210	1028	731	0	2344	8.9
	375	210	976.1	731	18.8	2311	8.6
	375	210	924.8	731	37.7	2278	8.2
	375	210	873.4	731	56.5	2246	7.6
	375	210	822.0	731	75.3	2213	6.8
	375	210	770.6	731	94.1	2181	5.8
	375	210	719.3	731	113.0	2148	4.5

**Table 4:** Change in the characteristics of concrete (∆) in % depending on the amount of mineral coarse aggregate replaced by walnut shell.

Conerete Characteristics	∆ in % with the Content of Coarse Walnut Shell Aggregate (%) by Volume of Coarse Aggregate						
	0	5	10	15	20	25	30
ρ (kg/m^3^)	0	–3	–5.3	–7.6	–10.0	–13.2	–15.1
*R*_b.cub_ (MPa)	0	2.7	–6.3	–19.6	–29.6	–35.4	–44.2
*R*_b_ (MPa)	0	3.3	–5.3	–23.1	–29.4	–35.0	–48.4
*R*_btb_ (MPa)	0	3.4	–6.8	–27.1	–37.3	–44.1	–47.5
*R*_bt_ (MPa)	0	3.3	–3.3	–16.7	–30.0	–33.3	–40.0

## Data Availability

The study did not report any data.

## Abbreviations

BD	Bulk Density
CCQ	Coefficient of Constructive Quality
CS	Compressive Strength
SC	Strength Characteristics
TD	True Density
TS	Tensile Strength
